# Chitosan and Cashew Nut Shell Liquid as Sustainable Additives: Enhancing Starch Digestibility and Reducing Methane Emissions in High-Grain Diets for Feedlot Cattle

**DOI:** 10.3390/polym17131860

**Published:** 2025-07-03

**Authors:** Raquel Tenório de Oliveira, Rafael Henrique de Tonissi e Buschinelli de Goes, Jefferson Rodrigues Gandra, Fernanda Naiara Fogaça da Cruz, Nayara Gonçalves da Silva, Lara de Souza Oliveira, Jaqueline Luiza Royer, Lucas Gabriel Batista Domiciano, Tainá Lorraine Pereira Azevedo, Carolina Marques Costa Araújo

**Affiliations:** 1 Faculty of Agricultural Sciences, Federal University of Grande Dourados, Dourados 79804-970, Brazil; raqueltennorio@gmail.com (R.T.d.O.); fogaca.fernandaa@gmail.com (F.N.F.d.C.); nayagsm@hotmail.com (N.G.d.S.); veterinarialaraoliveira@gmail.com (L.d.S.O.); jaqueline.royer123@gmail.com (J.L.R.); domicianolucasb@gmail.com (L.G.B.D.); carolinaaraujo@ufgd.edu.br (C.M.C.A.); 2 Faculty of Veterinary Medicine, Institute of Humid Tropic Studies, Federal University of the South and Southeast of Pará, Xinguara 68555-970, Brazil; jeffersongandra@unifesspa.edu.br; 3 Veterinary Medicine Department, State University of Maringá, Umuarama 87507-190, Brazil; tainalpazevedo@gmail.com

**Keywords:** methane, natural additives, ruminal fermentation, beef cattle

## Abstract

Chitosan and technical cashew nutshell liquid (CNSLt) have emerged as promising natural compounds due to their antimicrobial, immunomodulatory, and fermentation-modulating properties. This study aimed to evaluate the inclusion of chitosan and CNSLt as potential substitutes for the ionophore monensin on feed intake, ruminal fermentation, nitrogen balance, and microbial protein synthesis in steers. Five crossbred steers (*Bos taurus*), 18 months old with an average body weight of approximately 350 kg and fitted with permanent ruminal cannulas, were assigned to a 5 × 5 Latin square design. The experimental diets consisted of: (1) control (CON), (2) monensin (MON; 25 mg/kg of dry matter [DM]), (3) chitosan (CHI; ≥850 g/kg deacetylation degree, 375 mg/kg DM), (4) CNSLt (500 mg/kg DM), and (5) CNSLt + CHI (500 + 375 mg/kg DM). Supplementation with CHI or CNSLt + CHI reduced the intake of dry matter, crude protein, and neutral detergent fiber. Additionally, fecal excretion of whole corn kernels increased in these treatments. Ruminal fermentation parameters were affected, with the CNSLt + CHI treatment promoting higher molar proportions of propionate and acetate, along with reduced estimated methane emissions. However, purine derivatives, microbial protein synthesis, and nitrogen balance were not significantly affected by any of the treatments. These results suggest that CNSLt and CHI, particularly when combined, may serve as effective natural alternatives to monensin in high-grain diets for ruminants.

## 1. Introduction

A high-grain diet is commonly used in beef cattle to enhance weight gain and productivity. However, the increased availability of rapidly fermentable carbohydrates leads to elevated production of volatile fatty acids (VFAs) and the accumulation of lactic acid, which can result in subacute ruminal acidosis (SARA). The occurrence and persistence of SARA can disrupt the ruminal environment by causing the death of Gram-negative bacteria and promoting the proliferation of acid-tolerant bacteria that thrive at a ruminal pH significantly lower than the recommended level [1].

Alterations in ruminal fermentation can compromise the efficient utilization of diets. In this context, feed additives serve as an alternative to mitigate the risk of acidosis, improve dietary utilization efficiency, and enhance both animal performance and health [2,3]. Among the commonly used additives, ionophores such as monensin are widely applied. However, restrictions on the use of antimicrobials in animal production [4] and food safety regulations imposed by importing countries have prompted the search for alternatives to the use of ionophores [5].

Optimizing ruminal fermentation is crucial for improving feed efficiency and enhancing overall ruminant productivity. Traditional strategies, such as the use of ionophores (i.e., monesin and lasalocin), have been widely employed to regulate microbial populations and fermentation patterns. However, increasing regulatory restrictions and growing consumer concerns regarding antibiotic residues have intensified the search for alternative feed additives. Chitosan, a deacetylated derivative of crustacean chitin, has emerged as a promising natural compound due to its antimicrobial, immunomodulatory, and fermentation-enhancing properties [6]. Studies have shown that chitosan supplementation in ruminant diets can modify the ruminal microbiota, increase propionate production while reducing acetate and methane emissions [7,8]. Additionally, its ability to inhibit fibrinolytic bacteria and protozoa suggests a potential role in enhancing nitrogen utilization and reducing ammonia concentrations in the rumen [9].

Despite these promising results, the mechanisms by which chitosan interacts with ruminal microorganisms and fermentation processes remain not fully understood. Further research is needed to refine dosage strategies and evaluate the long-term effects of chitosan supplementation on animal performance, nutrient digestibility, and environmental sustainability.

Technical cashew nut shell liquid (CNSLt) has emerged as a promising natural additive with multiple benefits for ruminant nutrition and methane mitigation. Rich in phenolic compounds such as anacardic acid, cardanol, and cardol, CNSLt exhibits strong antimicrobial activity, selectively inhibiting methanogenic archaea while promoting propionate-producing microorganisms [10]. In vitro studies have shown that CNSLt supplementation can reduce methane emissions by up to 70.1% and increase propionate production by 44.4%, effectively shifting ruminal fermentation toward a more energy-efficient profile [10].

Additionally, CNSLt influences microbial populations by disrupting the cell membranes of hydrogen-producing bacteria, resulting in a more favorable fermentation pattern characterized by reduced acetate and butyrate production [11]. These findings underscore CNSLt’s potential as an environmentally sustainable alternative to conventional feed additives, such as ionophores, for improving ruminant productivity and reducing greenhouse gas emissions.

Based on this evidence, we hypothesized that the combined supplementation of chitosan and technical cashew nutshell liquid (CNSLt) would enhance ruminal fermentation and digestion without negatively affecting feed intake, nitrogen balance, or microbial protein synthesis in high-grain diets for feedlot cattle. Therefore, the objectives of this study were to evaluate the effects of chitosan and CNSLt, individually and in combination, on ruminal fermentation, nutrient digestion, and microbial protein synthesis. Additionally, this study aimed to assess the impact of these additives on feed intake and nitrogen balance, supporting their potential as sustainable alternatives to conventional feed additives in high-grain diets.

## 2. Materials and Methods

### 2.1. Location, Animals, and Treatments

The experiment was conducted at the Ruminant Nutrition Sector, Animal Nutrition Laboratory, and By-Product Evaluation Laboratory (LAPAC/FINEP) of the Federal University of Grande Dourados (UFGD), Brazil.

Five crossbred steers (Holstein × Zebu), aged 18 months, castrated, with an average body weight of approximately 350 kg, and fitted with permanent ruminal cannulas, were used. The animals were housed in individual covered pens (24 m^2^; 4 × 6 m) with concrete flooring, each equipped with individual feed troughs and automatic waterers with a supply of 60 L/animal/day. A 5 × 5 Latin square design was employed, with animals randomly assigned to treatments across five experimental periods. Each period lasted 19 days, including 10 days of dietary adaptation and 9 days of data collection.

The diet consisted of 85% corn grain and 15% protein pellet, formulated to meet the nutritional requirements of growing steers. Treatments were based on the inclusion of chitosan (CHI) [12] and technical cashew nut shell liquid (CNSLt) [13], either alone or in combination. Chitosan (≥850 g/kg deacetylation degree, 0.32 g/mL density, pH 7.90, viscosity) was supplied by Polymar Indústria e Comércio de Importação e Exportação LTDA (Fortaleza, CE, Brazil), and CNSLt was obtained from Usibras Company (Aquiraz, CE, Brazil). The CNSLt used contained 10.03 mg/g of anacardic acid, 540.77 mg/g of cardanol, 102.34 mg/g of cardol, and 19.17 mg/g of 2-methylcardol, and its composition was confirmed by chemical analysis [13]. The experimental treatments were as follows: CON: control diet without additives; MON: monensin at 25 mg/kg of dry matter (DM); CHI: chitosan at 375 mg/kg DM; CNSLt: CNSLt at 500 mg/kg DM; and CNSLt + CHI: combination of CNSLt (500 mg/kg DM) and CHI (375 mg/kg DM).

Animals were fed one time daily (08:00), based on the previous day’s dry matter intake. Feed offered and refusals (orts) were recorded daily, maintaining a 5–10% surplus to avoid feed restriction. The two dietary components (whole corn and the pellet) were manually mixed in the feed trough and offered as a total mixed ration (TMR) (Table 1).

### 2.2. Nutrient Intake and Apparent Total Digestibility

Dry matter intake (DMI) was determined by calculating the difference between the amount of feed offered and feed refused (orts), and it was also estimated based on total fecal dry matter (DM) excretion. To estimate fecal DM output, titanium dioxide (TiO_2_) was used as an external marker. TiO_2_ was administered daily in paper cartridges at a dosage of 5 g/day and introduced directly into the rumen via the cannula once daily at 08:00 for 10 consecutive days. The initial five days were designated for external marker adaptation, while fecal sampling occurred over the following five days [14].

Beginning on the seventh day of each experimental period, fecal samples (~200 g) were collected directly from the rectal ampulla at multiple time points (08:00, 10:00, 12:00, 14:00, and 16:00). Samples were stored in labeled plastic trays and pre-dried in a forced-air oven at 55 °C. At the end of each period, samples from each animal were pooled to form a composite sample per period. Fecal TiO_2_ concentrations were determined by UV/Vis spectrophotometry (380 nm), as described by [15].

Fecal excretion was estimated using the following equation:

$$\mathrm{FE}=\frac{\mathrm{FI}}{\mathrm{TFC}}$$

where FE = daily fecal excretion (g/day), FI = titanium dioxide intake (g/day), and TFC = titanium dioxide concentration in feces (g/g DM).

Apparent total digestibility coefficients were determined for dry matter (DM), crude protein (CP), and organic matter (OM). Analyses were conducted following standard AOAC procedures: DM (method 930.15), CP (N × 6.25; method 984.13), and ash (ASH; method 942.05), with OM calculated as OM = 100−ASH [16]. Fiber fractions, including neutral detergent fiber (NDF) and acid detergent fiber (ADF), were analyzed according to Van Soest et al. [17]. Starch content was measured using the enzymatic colorimetric method described by [18].

To quantify corn grain excretion in feces, samples were collected directly from the rectal ampulla of each animal between 09:00 and 11:00 on the 16th day of each experimental period. A 300 g fecal sample was weighed using an analytical balance and washed under running water through a 2.00 mm sieve (Granutest, Tyler 9, ABNT 10, Prolab, São Paulo, Brazil). The recovered corn grain particles were manually collected, weighed, and dried in a forced-air oven at 55 °C for 72 h to determine their dry matter content [19].

### 2.3. Ruminal Fermentation

On the 19th day of each experimental period, ruminal fluid samples were manually collected to determine ruminal pH, ammonia nitrogen (N-NH_3_) concentration, short-chain fatty acid (SCFA), and branch-chain fatty acid (BCFA) profiles. Sampling occurred immediately before feeding and at 2, 4, 6, and 8 h post-feeding, with samples obtained from the liquid–solid interface of the rumen. The collected samples were filtered through triple-layered gauze. Ruminal pH was measured using a portable digital pH meter (Meta Química, Meta 210P, São Paulo, Brazil).

For SCFA analysis, 20 mL of ruminal fluid was centrifuged at 3500 rpm for 5 min. From the supernatant, 1800 µL was mixed with 100 µL of 20% ortho-phosphoric acid and frozen for later analysis. Additionally, 1600 µL aliquots were mixed with 400 µL of formic acid (98–100%) and centrifuged at 7000× *g* for 15 min at 4 °C.

SCFA concentrations were determined using a gas chromatograph (GC-2010 Plus, Shimadzu, Barueri, Brazil) equipped with an automatic injector (AOC-20i), a Stabilwax-DA™ capillary column (30 m, 0.25 mm ID, 0.25 µm df; Restek^®^, Bellefonte, PA, USA), and a flame ionization detector. Samples were acidified with 1 M ortho-phosphoric acid (Merck^®^, Rahway, NJ, USA, Ref. 100573) and fortified with a mixture of free volatile acids (Supelco^®^, St. Louis, MO, USA, Ref. 46975). A 1 µL aliquot was injected with a split ratio of 40:1, using helium as the carrier gas at a linear velocity of 42 cm·s^−1^. The total chromatographic run time was 11.5 min. The injector and detector temperatures were set at 250 °C and 300 °C, respectively. The column temperature program began at 40 °C, ramping to 120 °C at 40 °C/min, to 180 °C at 10 °C/min, and finally to 240 °C at 120 °C/min, where it was held for 3 min. Quantification was performed using dilutions of the WSFA-2 standard (Supelco^®^, Ref. 47056) and glacial acetic acid (Sigma-Aldrich^®^, St. Louis, MO, USA, Ref. 33209), with peak identification and integration conducted using GCsolution v.2.42.00 software (Shimadzu^®^).

For N-NH_3_ analysis, 40 mL of ruminal fluid was preserved with 1 mL of 1:1 HCl and frozen at −18 °C. Ammonia nitrogen concentration was determined via distillation using 2N KOH as the distillation base, following prior centrifugation at 1000× *g* for 15 min, without acid digestion [20].

Methane production (mM/L) was estimated using the following equation [21]:

$${\mathrm{CH}}_{4}=0.45\left(C_{2}\right)-0.275\left(C_{3}\right)+0.40\left(C_{4}\right)$$

where C_2_, C_3_, and C_4_ represent acetate, propionate, and butyrate concentrations (mM), respectively.

### 2.4. Microbial Protein Synthesis

Urine samples were collected on the 15th, 16th, 17th, and 18th day of each experimental period using the spot sampling method during spontaneous urination, 4 h after supplement feeding [22]. To determine concentrations of creatinine, urea, uric acid, and allantoin, a 10 mL urine aliquot was diluted in 40 mL of 0.036 N sulfuric acid. An additional 40 mL aliquot was preserved in 1 mL of concentrated sulfuric acid (36 N) for total urinary nitrogen (N) analysis. All samples were properly labeled and stored at −18 °C.

Allantoin concentration was determined using a colorimetric method [23,24]. Commercial assay kits (Labtest^®^, Lagoa Santa, Brazil; Gold Analisa^®^ Diagnóstica Ltd.a, Belo Horizonte, Brazil) were used to quantify creatinine, urea, and uric acid concentrations.

The total excretion of purine derivatives (PD, mmol/day) was calculated by summing urinary allantoin and uric acid. The absorbed microbial purines (Pabs, mmol/day) were estimated using the following equation [25]:

$$\mathrm{PD}=0.85*\;\mathrm{Pabs}+0.385{\;*\mathrm{BW}}^{0.75}$$

where BW is the body weight of the animal in kilograms.

Daily excretion of urea nitrogen (N-urea) and creatinine nitrogen (N-creatinine) was calculated by multiplying the concentrations of urea and creatinine in the spot urine sample by the estimated 24 h urinary volume, using correction factors of 0.466 and 0.3715, respectively, which correspond to the nitrogen content of urea and creatinine.

The estimated daily urinary volume (UV, L/day) was calculated using the following equation [26]:

$$\mathrm{UV}=\frac{27.36*\mathrm{BW}}{\left[\mathrm{Creatinine}\right]}$$

where BW is the animal’s body weight (kg), and [Creatinine] is the creatinine concentration (mg/L) in the spot urine sample. The constant 27.36 represents the average daily creatinine excretion (mg/kg BW/day) for crossbred and Zebu steers. Microbial protein synthesis = (Urinary excretion of purine derivatives) x (a constant value) [26].

Nitrogen balance (NB) was calculated as the difference between total nitrogen intake and total nitrogen excretion in urine and feces. Nitrogen concentrations in urine and feces were determined using the micro-Kjeldahl method. Retained nitrogen (NRet) was estimated by subtracting Absorbed N–Urine N.

### 2.5. Urea and Creatinine Metabolism

On the 17th day of each experimental period, blood samples were collected from the caudal vein four hours after feeding. Heparin was used as an anticoagulant. Immediately after collection, the samples were centrifuged at 5000 rpm for 15 min to separate the plasma. The supernatant was then labeled and stored at −18 °C until analysis. Plasma urea and creatinine concentrations were determined using a commercial enzymatic assay kit (Gold Analisa^®^ Diagnóstica Ltda, Belo Horizonte, Brazil).

### 2.6. Statistical Analysis

Data were analyzed using the MIXED procedure of SAS (Statistical Analysis System, version 9.4, SAS Institute Inc., Cary, NC, USA) in a 5 × 5 Latin square design, considering fixed effects of treatment, period, and random effects of animal and residual error, with the following model:

Y_ijl_ = μ + A_i_ + P_j_ + D_l_ + err_ijl_

where Y_ijl_ = dependent variable; μ = overall mean; A_i_ = animal effect (i = 1 to 5); P_j_ = period effect (j = 1 to 5); D_l_ = diet effect; and e_ijl_ = experimental error.

Repeated measures over time (for variables such as pH, N-NH_3_, and SCFA) were analyzed using the REPEATED statement, and the best covariance structure was selected based on the lowest Akaike Information Criterion (AIC) value, according to the following model:

Y_ijk_ = μ + A_i_ + P_j_ + D_k_ + T_y_ + T_y_(D_k_) + err_ijk_

where Y_ijk_ = dependent variable; μ = overall mean; A_i_ = animal effect (i = 1 to 5); P_j_ = period effect (j = 1 to 5); D_k_ = treatment effect (k = 1 to 5); T_y_ = time effect (y = 1 to 5); T_y_ (D_k_) = interaction between diet and time; and err_ijk_ = experimental error.

Treatment means were compared using Tukey’s test at a significance level of *p* < 0.05. Data normality and homoscedasticity were verified using the Shapiro–Wilk and Levene’s tests, respectively. Results are presented as least squares means ± standard error of the mean (SEM).

## 3. Results

Steers supplemented with CHI + CNSLt exhibited lower (*p* ≤ 0.032) intakes of dry matter (DM), corn kernels, pellets, organic matter (OM), crude protein (CP), and neutral detergent fiber (NDF) compared to those fed the CON, CNSLt, and MON diets (Table 2). However, no significant differences were observed in comparison to steers supplemented with CHI alone. Additionally, CHI-supplemented steers did not differ from those on the other experimental diets.

Steers supplemented with CHI + CNSLt exhibited greater (*p* = 0.008) starch digestibility compared to those receiving the CON, CNSLt, and MON diets. However, no significant differences were observed in comparison to steers supplemented with CHI alone.

Additionally, CHI + CNSLt supplementation resulted in lower (*p* = 0.012) corn kernel excretion in feces compared to steers receiving the CON and CNSLt treatments, with no significant differences observed relative to CHI or MON.

Furthermore, CHI + CNSLt-supplemented steers showed lower (*p* = 0.014) ruminal ammonia nitrogen concentrations compared to all other treatment groups. Regarding short-chain fatty acid concentrations, steers supplemented with CHI + CNSLt exhibited lower (*p* ≤ 0.045) acetate and butyrate concentrations, higher (*p* = 0.038) propionate concentrations, and consequently lower (*p* = 0.018) estimated methane concentrations compared to the other treatment groups (Table 3).

Additionally, steers receiving CHI or CNSLt individually showed higher (*p* ≤ 0.045) concentrations of acetate, butyrate, and methane than those in the other groups. Steers supplemented with MON exhibited intermediate (*p* ≤ 0.045) concentrations of acetate, propionate, butyrate, and methane compared to those receiving CHI, CNSLt, or CHI + CNSLt.

Steers supplemented with CHI + CNSLt also demonstrated lower (*p* = 0.027) nitrogen intake than those receiving the CON, CNSLt, and MON diets (Table 4); however, no significant differences were observed when compared to steers supplemented with CHI alone.

Additionally, steers supplemented with CHI + CNSLt exhibited intermediate nitrogen retention (*p* = 0.007) compared to the other groups. Steers supplemented with CNSLt alone showed the highest nitrogen retention, whereas those receiving CHI alone presented the lowest values.

No significant differences were observed among treatments regarding microbial protein synthesis (Table 4), as well as urea and creatinine metabolism parameters (Table 5).

## 4. Discussion

Our hypothesis proposed that combined supplementation with chitosan and cashew nutshell liquid (CNSL) would enhance ruminal fermentation and digestion without adversely affecting feed intake, nitrogen balance, or microbial protein synthesis in high-grain diets for feedlot cattle. The results provide partial support for this hypothesis. Supplementation with CHI + CSNLt improved starch digestibility and altered ruminal fermentation patterns, evidenced by increased propionate concentrations and reduced levels of acetate, butyrate, and methane. These changes suggest a shift toward more efficient energy utilization within the rumen.

Additionally, the reduced fecal excretion of corn kernels suggests enhanced starch utilization. However, contrary to expectations, nitrogen retention in the CHI + CSNLt group was intermediate, with CSNLt alone resulting in the highest retention and CHI alone the lowest. Furthermore, microbial protein synthesis and urea and creatinine metabolism remained unaffected, suggesting that CHI + CSNLt supplementation did not significantly enhance nitrogen utilization as anticipated. While the combined additives improved digestion and fermentation efficiency, their effects on nitrogen metabolism and microbial protein synthesis were less pronounced. Therefore, the hypothesis is supported primarily regarding fermentation and digestibility improvements, warranting further investigation into their influence on nitrogen metabolism and protein dynamics in high-concentrate diets.

The observed reduction in dry matter, organic matter, and fiber intake in CHI + CSNLt-supplemented steers may be attributed to metabolic regulation of intake, particularly influenced by increased ruminal propionate concentrations [27,28,29]. Propionate, a key gluconeogenic volatile fatty acid (VFA), contributes to satiety signaling via hepatic oxidation, potentially resulting in earlier satiety and reduced intake [30].

The improved total starch digestibility and decreased fecal corn kernel excretion suggest enhanced ruminal starch degradation and post-ruminal absorption, possibly linked to the antimicrobial effects of chitosan and CNSL [12]. These additives may modulate ruminal microbial populations by promoting amylolytic bacteria while suppressing excessive proteolysis and methanogenesis. The shift toward higher propionate and lower acetate and butyrate levels supports this mechanism.

Despite reduced intake, the greater energy yield from increased propionate availability may have compensated for lower energy intake, maintaining the energy supply. However, the absence of significant differences between CHI + CSNLt and CHI alone suggests that chitosan may have had a predominant role in these metabolic effects, while CNSL may have offered an additive, but not synergistic, benefit for fermentation and digestive efficiency.

The physical form of the diet may have also supported ruminal pH maintenance. Whole corn kernels digest more slowly than processed corn, and pellets provide a source of NDF that supports ruminal motility, saliva production, and pH buffering [12]. The high propionate concentration typical of corn-based diets [31] was likely further increased by chitosan’s ability to shift fermentation toward propionate production through inhibition of Gram-positive bacteria [32,33]. The combination of CHI and CNSLt may have intensified this effect, as anacardic acid in CNSLt has been reported to enhance propionic acid production [10].

The reduction in ruminal ammonia nitrogen concentrations in steers supplemented with CHI + CSNLt may reflect the modulation of proteolytic microbial activity. Both chitosan and CNSLt selectively inhibit Gram-positive bacteria, including hyper-ammonia-producing species, thereby potentially reducing deamination rates and improving nitrogen use efficiency [34].

The changes in SCFA profiles—reduced acetate and butyrate alongside elevated propionate—further support the occurrence of microbial modulation. Enhanced starch digestibility and lower fecal starch loss in the CHI + CSNLt group are consistent with this shift toward greater fermentation efficiency and energy utilization [35]. Additionally, the decrease in methane concentration may be due to the propionate-promoting effects of the additives, as propionate serves as a competitive hydrogen sink, limiting substrate availability for methanogenesis [10]. The intermediate fermentation profile in MON-supplemented steers suggests only partial modulation, while the elevated acetate, butyrate, and methane levels in CHI- and CSNLt-supplemented groups suggest that the additives alone were less effective in promoting a glucogenic profile compared to their combined use.

The CHI + CSNLt combination demonstrated effects on ruminal fermentation similar to those of ionophores [35,36]. The observed reduction in acetate likely reflects the inhibition of Gram-positive acetate-producing bacteria, promoting propionate synthesis [32,33]. The VFA profile in the rumen is influenced by diet composition and microbial populations, and high-starch diets, such as those used in this study, favor succinate- and propionate-producing bacteria [37].

Reduced methane production with CHI + CSNLt supplementation may involve several mechanisms. Chitosan has been shown to reduce H_2_ ion concentrations and inhibit methanogenic bacteria [32], while anacardic acids in CNSL exert strong anti-methanogenic effects [38]. Additionally, by enhancing propionate production, CNSL indirectly reduces hydrogen availability for methanogenesis, further decreasing CH_4_ emissions [13,34].

The reduction in methane production observed with CHI + CSNLt supplementation may be associated with the combined mechanisms of action of these additives. Chitosan creates a ruminal environment with lower H_2_ ion concentrations and exerts antimicrobial activity against methanogenic bacteria [32]. Meanwhile, CNSLt contains anacardic acids, which have a significant inhibitory effect on methanogenic bacteria [38]. Furthermore, CNSLt enhances propionate production, and this VFA competes directly with methane for the available H_2_, thereby reducing hydrogen availability for methanogenesis [13,34].

The lower nitrogen intake in the CHI + CSNLt group was consistent with the reduced dry matter and crude protein intake, suggesting feed intake regulation by these additives. Despite this, the intermediate nitrogen retention observed may suggest a tendency toward improved nitrogen utilization efficiency [13,35]. This is supported by the reduced ruminal ammonia concentrations in this group, which suggests lower proteolysis and deamination rates, likely due to suppression of hyper-ammonia-producing microbes. Enhanced starch digestibility may have promoted a more synchronized energy-to-nitrogen ratio, favoring microbial protein synthesis [9].

The greater nitrogen retention observed in steers supplemented with CSNLt alone may reflect moderate modulation of fermentation, allowing for more effective microbial nitrogen capture. In contrast, lower retention in CHI-supplemented animals may suggest that its antimicrobial action disrupted microbial protein synthesis or altered nitrogen recycling dynamics [9].

In summary, CHI + CSNLt supplementation significantly modulated ruminal fermentation by promoting a glucogenic profile with increased propionate production and reduced acetate, butyrate, and methane emissions. These changes, along with improved starch digestibility and lower fecal starch loss, highlight the potential of CHI + CSNLt as an effective strategy to optimize fermentation and feed efficiency in high-starch, low-fiber diets. Although the effects on nitrogen metabolism were less pronounced, the combination showed promise in improving nitrogen utilization efficiency.

Future studies should investigate the long-term effects of CHI + CSNLt supplementation on performance, microbial community dynamics, and metabolic health. Further exploration into dose optimization, interactions with various starch sources, and impacts on post-ruminal digestibility and systemic metabolism may enhance its practical application in intensive feedlot systems.

## 5. Conclusions

The combined supplementation of chitosan and cashew nut shell liquid (CHI + CSNLt) proved to be an effective strategy for modulating ruminal fermentation in high-grain diets. It enhanced starch digestibility, reduced methane emissions, and optimized nitrogen utilization. These findings indicate that CHI + CSNLt may serve as a viable alternative to monensin sodium, particularly in diets characterized by high levels of fermentable starch and low physically effective fiber, where metabolic disorders such as subacute ruminal acidosis are of concern.

By promoting a more glucogenic fermentation profile while simultaneously mitigating nitrogen losses and enteric methane production, CHI + CSNLt emerges as a promising and sustainable feed additive for intensive feedlot systems. Nonetheless, further research is needed to determine optimal dosing strategies, assess long-term effects on the ruminal microbiome, and evaluate the impacts on overall animal performance and health. Despite these considerations, under extreme dietary conditions, the CHI + CSNLt combination presents a compelling alternative to ionophores, offering both performance advantages and potential improvements in the environmental sustainability of ruminant production.

## Figures and Tables

**Table 1:** Ingredient proportions and chemical composition of the supplements utilized.

Ingredients		(g/kg de MS)	
Whole corn		850	
Protein, vitamin, and mineral pellet ^1^		150	
	Chemical composition		
g/kg DM	Corn	Pellet	Diet
Dry matter	850	912.1	845.8
Organic matter	957	822.5	933.2
Crude protein	95	394.7	137
Neutral detergent fiber	96	372.9	241
Acid detergent fiber	18	236.9	63.5
Starch	750	38.9	651
Total digestible nutrients	797.86	682.36	737.38

^1^ Ca, 43 g/kg; P, 10 g/kg; S, 4 g/kg; Mg, 0.7 g/kg; K, 2.7 g/kg; Na, 9.7 g/kg; Co, 5 mg/kg; Cu, 175 mg/kg; Cr, 1.4 mg/kg; F, 130 mg/kg; I, 5 mg/kg; Mn, 182 mg/kg; Mo, 0.35 mg/kg; Zn, 421 mg/kg; Vitamin A, 21.000 U.I; Vitamin D, 3.000 U.I; Vitamin E, 140 U.I.

**Table 2:** Intake and digestibility according to experimental diets.

Item	Experimental Diets ^1^					SEM ^2^	*p*-Value
	CON	CHI	CSNLt	CHI + CSNLt	MON		
Intake (g/d)							
Dry matter	7.46 ^a^	7.23 ^ab^	8.09 ^a^	6.60 ^b^	7.41 ^a^	0.387	0.032
Dry matter, %BW	2.24	1.89	2.10	2.26	2.20	0.122	0.554
Corn kernel	6.34 ^ab^	6.15 ^b^	6.87 ^a^	5.61 ^b^	6.29 ^ab^	0.329	0.017
Pellet	1.12 ^ab^	1.08 ^b^	1.21 ^a^	0.990 ^b^	1.11 ^ab^	0.058	0.026
Organic matter	7.16 ^a^	6.95 ^ab^	7.77 ^a^	6.34 ^b^	7.11 ^a^	0.376	0.018
Crude protein	0.984 ^a^	0.954 ^ab^	1.06 ^a^	0.872 ^b^	0.984 ^a^	0.051	0.026
NDF	2.45 ^a^	2.32 ^ab^	2.83 ^a^	1.94 ^b^	2.42 ^a^	0.231	0.017
Starch	3.86 ^a^	3.71 ^ab^	3.27 ^a^	3.30 ^b^	3.82 ^a^	0.387	0.032
Digestibility (g/kg)							
Dry matter	534	454	504	568	504	4.194	0.124
Organic matter	568	488	530	596	535	4.398	0.147
Crude protein	700	616	686	718	702	5.470	0.149
NDF	486	404	456	502	445	7.347	0.161
Starch	792 ^b^	826 ^a^	806 ^b^	854 ^a^	815 ^b^	3.143	0.008
Excretion of intact corn kernel (g/kg)							
Corn kernel	306.5 ^a^	268.2 ^ab^	301.6 ^a^	241.6 ^b^	276.7 ^ab^	2.985	0.012

^1^ CON (no additive); CHI (supplemented with 375 mg/kg DM); CNSLt (supplemented with 500 mg/kg DM of technical cashew nutshell liquid); CHI + CNSLt (supplemented with 375 mg/kg DM of chitosan + 500 mg/kg DM of technical cashew nutshell liquid) and MON (supplemented with 25 mg/kg DM of sodic monensin). ^2^ SEM (standard error of mean). ^a,b^ Means followed by different letters on the same line differ by 5% in the TUKEY test adjusted by SAS PROC MIXED.

**Table 3:** Ruminal fermentation according to experimental diets.

Item	Experimental Diets ^1^					SEM ^2^	*p*-Value
	CON	CHI	CSNLt	CHI + CSNLt	MON		
pH	6.21	6.22	6.20	6.28	6.28	0.006	0.654
N-NH_3_, mg/dL	20.96 ^a^	25.82 ^a^	23.83 ^a^	15.14 ^b^	22.61 ^a^	0.026	0.014
	mmol/L						
Acetate	44.56 ^b^	57.94 ^a^	54.37 ^a^	37.75 ^c^	47.53 ^b^	0.120	0.045
Propionate	30.16 ^a^	26.14 ^ab^	24.57 ^b^	33.51 ^a^	26.23 ^ab^	0.131	0.038
Butyrate	13.72 ^b^	19.04 ^a^	20.25 ^a^	10.65 ^c^	11.71 ^bc^	0.087	0.012
Isobutyrate	1.18	1.23	1.40	1.03	1.21	0.018	0.236
Isovalerate	4.05	4.37	3.66	3.07	4.61	0.040	0.414
Valerate	1.97	1.65	2.07	3.10	2.48	0.042	0.447
Brach chain fatty acids	7.20	7.24	7.13	7.19	8.30	0.046	0.484
Total	95.64	110.36	106.32	89.11	93.76	0.173	0.784
Acetate/propionate	1.93	2.24	2.47	1.79	2.06	0.028	0.654
Methane	17.25 ^ab^	25.81 ^a^	26.50 ^a^	12.03 ^b^	18.86 ^ab^	0.097	0.018

^1^ CON (no additive); CHI (supplemented of 375 mg/kg DM); CNSLt (supplemented of 500 mg/kg DM of technical cashew nutshell liquid); CHI + CNSLt (supplemented of 375 mg/kg DM of chitosan + 500 mg/kg DM of technical cashew nutshell liquid) and MON (supplemented of 25 mg/kg DM of sodic monensin). ^2^ SEM (standard error of mean). ^a–c^ Means followed by different letters on the same line differ by 5% in the TUKEY test adjusted by SAS PROC MIXED.

**Table 4:** Nitrogen balance and microbial protein synthesis according to experimental diets.

Item	Experimental Diets ^1^					SEM ^2^	*p*-Value
	CON	CHI	CSNLt	CHI + CSNLt	MON		
g/d							
N-intake	157.44 ^a^	152.64 ^ab^	169.60 ^a^	139.42 ^b^	156.26 ^a^	0.227	0.027
N-feces	16.13	21.68	25.63	14.23	19.53	0.149	0.224
N-urine	21.40	27.37	12.49	13.66	6.08	0.132	0.324
N-absorbed	141.31	130.96	143.97	125.19	136.73	0.242	0.442
N-retained	119.91 ^b^	103.59 ^c^	131.48 ^a^	111.53 ^b^	130.65 ^a^	0.246	0.007
mmol/d							
Allantoin	221.42	162.40	221.64	201.87	202.18	0.423	0.841
Uric acid	47.43	14.13	29.26	35.23	55.46	0.207	0.751
Total purines	268.81	176.54	250.84	237.09	257.64	0.443	0.801
Purines absorbed	303.45	193.47	280.71	267.42	198.37	0.482	0.872
g/d							
Microbial nitrogen	220.62	140.66	204.09	194.43	183.77	0.411	0.847
Microbial protein	1378.90	879.14	1275.54	1215.18	1198.59	1.028	0.847

^1^ CON (no additive); CHI (supplemented of 375 mg/kg DM); CNSLt (supplemented of 500 mg/kg DM of technical cashew nutshell liquid); CHI + CNSLt (supplemented of 375 mg/kg DM of chitosan + 500 mg/kg DM of technical cashew nutshell liquid) and MON (supplemented of 25 mg/kg DM of sodic monensin). ^2^ SEM (standard error of mean). ^a–c^ Means followed by different letters on the same line differ by 5% in the TUKEY test adjusted by SAS PROC MIXED.

**Table 5:** Urea and creatinine metabolism according to the experimental diets.

Item	Experimental Diets ^1^					SEM ^2^	*p*-Value
	CON	CHI	CSNLt	CHI + CSNLt	MON		
Urine (mg/dL)							
Urea	801.28	813.02	834.42	802.35	793.49	28.56	0.357
Creatinine	1.42	1.86	1.78	1.77	2.43	0.12	0.328
N-urea	352.69	354.35	361.64	355.97	338.55	12.98	0.325
N-Creatinine	0.529	0.746	0.802	0.722	0.688	0.015	0.357
Blood (mg/dL)							
Urea	20.75	20.79	22.59	23.22	24.08	1.88	0.335
Creatinine	2.76	2.57	2.00	2.31	2.92	0.74	0.247
N-urea	9.60	9.35	10.92	10.88	10.15	1.05	0.635
N-Creatinine	1.19	1.12	1.18	1.13	1.16	0.32	0.345
Excretion (mg/kg BW)							
Urea	887.58	820.64	783.50	889.66	669.22	15.89	0.868
Creatinine	27.47	27.75	27.41	27.53	27.51	3.21	0.865
Clearance (mg/kg BW)							
Urea	45.45	44.85	42.00	43.96	44.00	2.65	0.881
Creatinine	5.95	6.07	6.06	5.98	6.25	1.23	0.865
Fractional excretion (%)							
Urea	69.95	67.78	66.23	68.23	67.18	4.88	0.885

^1^ CON (no additive); CHI (supplemented of 375 mg/kg DM); CNSLt (supplemented of 500 mg/kg DM of technical cashew nutshell liquid); CHI + CNSLt (supplemented of 375 mg/kg DM of chitosan + 500 mg/kg DM of technical cashew nutshell liquid) and MON (supplemented of 25 mg/kg DM of sodic monensin). ^2^ SEM (standard error of mean).

## Data Availability

The original contributions presented in this study are included in the article. Further inquiries can be directed to the corresponding authors.
